# Supplementary material for: A Single Intranasal Dose of Bacterial Therapeutics to Calves Confers Longitudinal Modulation of the Nasopharyngeal Microbiota: a Pilot Study
Source: mSystems. 2023 Mar 27;8(2):e01016-22. doi: 10.1128/msystems.01016-22 (PMC10134831; doi:10.1128/msystems.01016-22)
Supplement: TABLE S1 [file msystems.01016-22-s0001.docx]

**Supplementary Table 1**

| Animal ID | Treatment group | Rectal temperature (°C ) | Treatment  Date | Antibiotic treatment | # of antibiotic treatments | Day of experiment |
| --- | --- | --- | --- | --- | --- | --- |
| 60 | BT | 40.1 | 19/11/18 | Micotil | 1 | 4 |
| 2 | BT | 39.7 | 20/11/18 | Micotil | 1 | 5 |
| 1 | CTRL | 40.6 | 17/18/11 | Micotil | 1 | 2 |
| 36 | CTRL | 39.8 | 19/11/18 | Micotil | 1 | 4 |
| 6 | CTRL | 39.7 | 4/12/19 | Micotil | 1 | 18 |
