# Supplementary figures and images for: A Single Intranasal Dose of Bacterial Therapeutics to Calves Confers Longitudinal Modulation of the Nasopharyngeal Microbiota: a Pilot Study
Source: mSystems. 2023 Mar 27;8(2):e01016-22. doi: 10.1128/msystems.01016-22 (PMC10134831; doi:10.1128/msystems.01016-22)

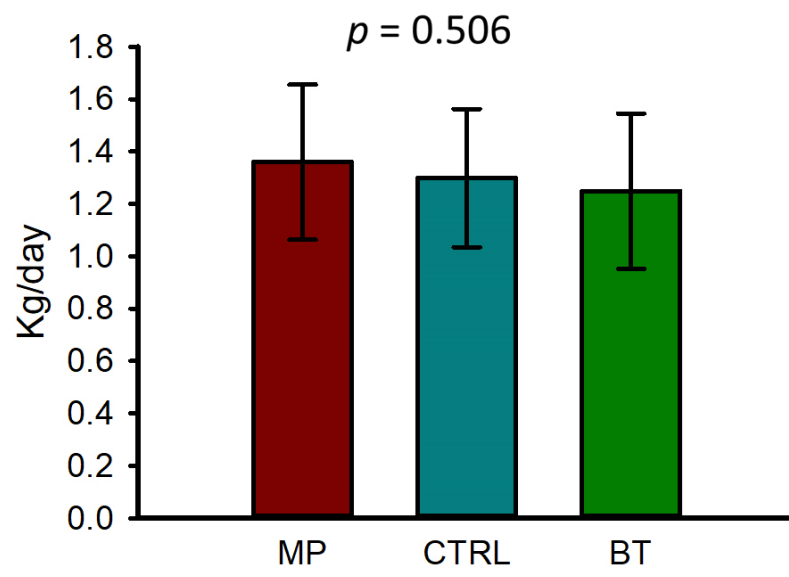

Supplement: FIG S1 [file msystems.01016-22-s0004.pdf]

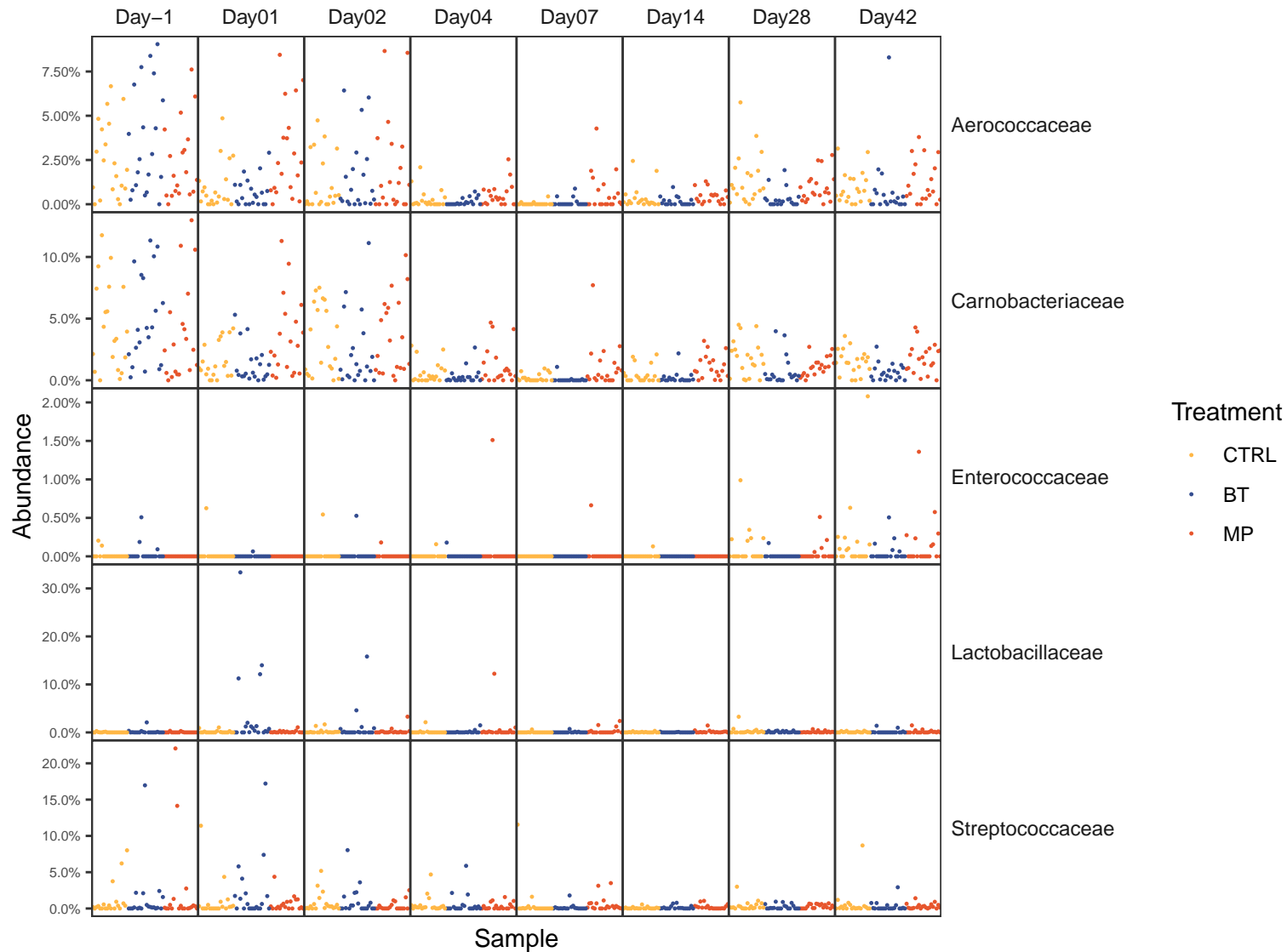

Supplement: FIG S2 [file msystems.01016-22-s0005.pdf]

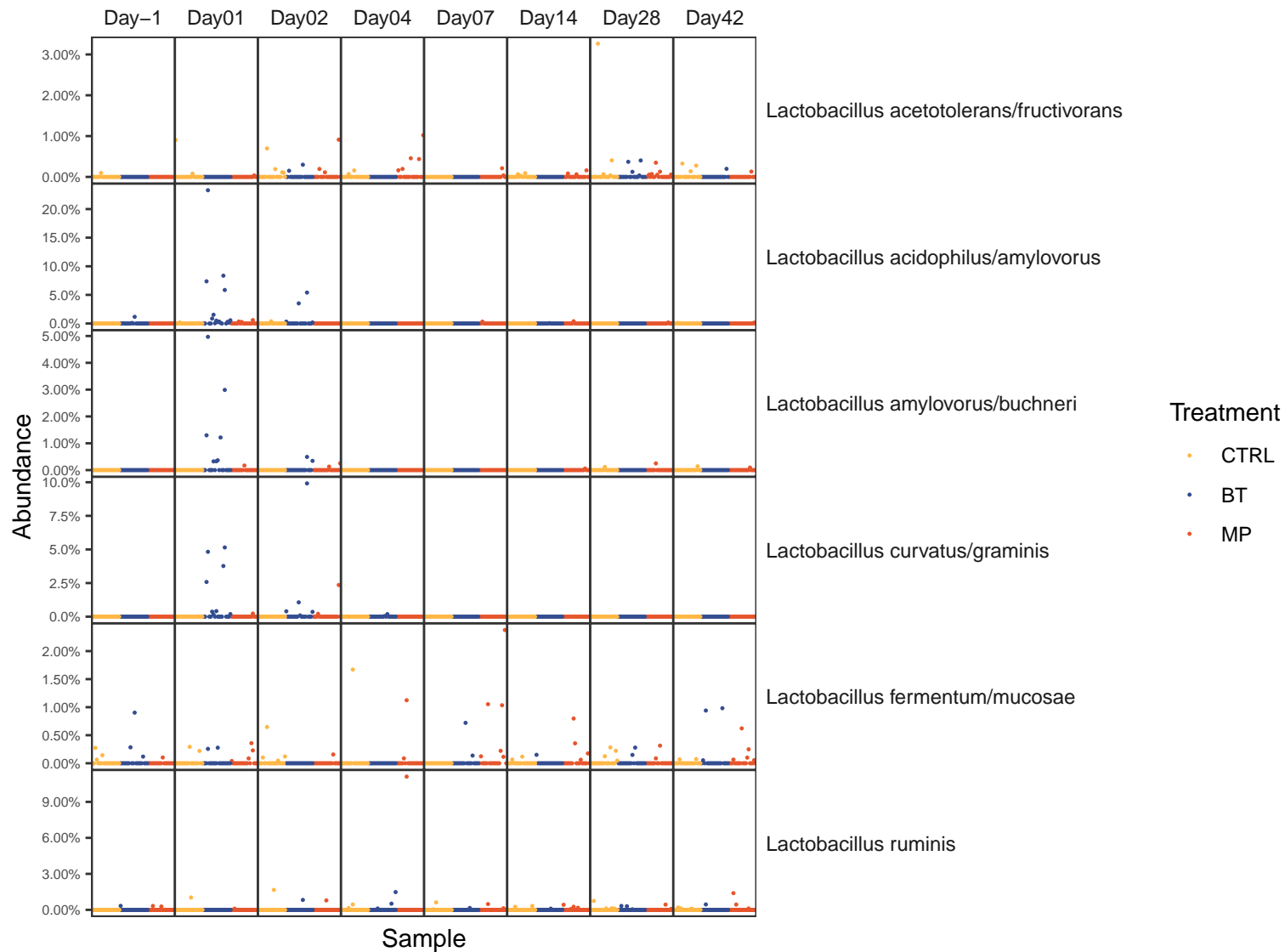

Supplement: FIG S3 [file msystems.01016-22-s0006.pdf]

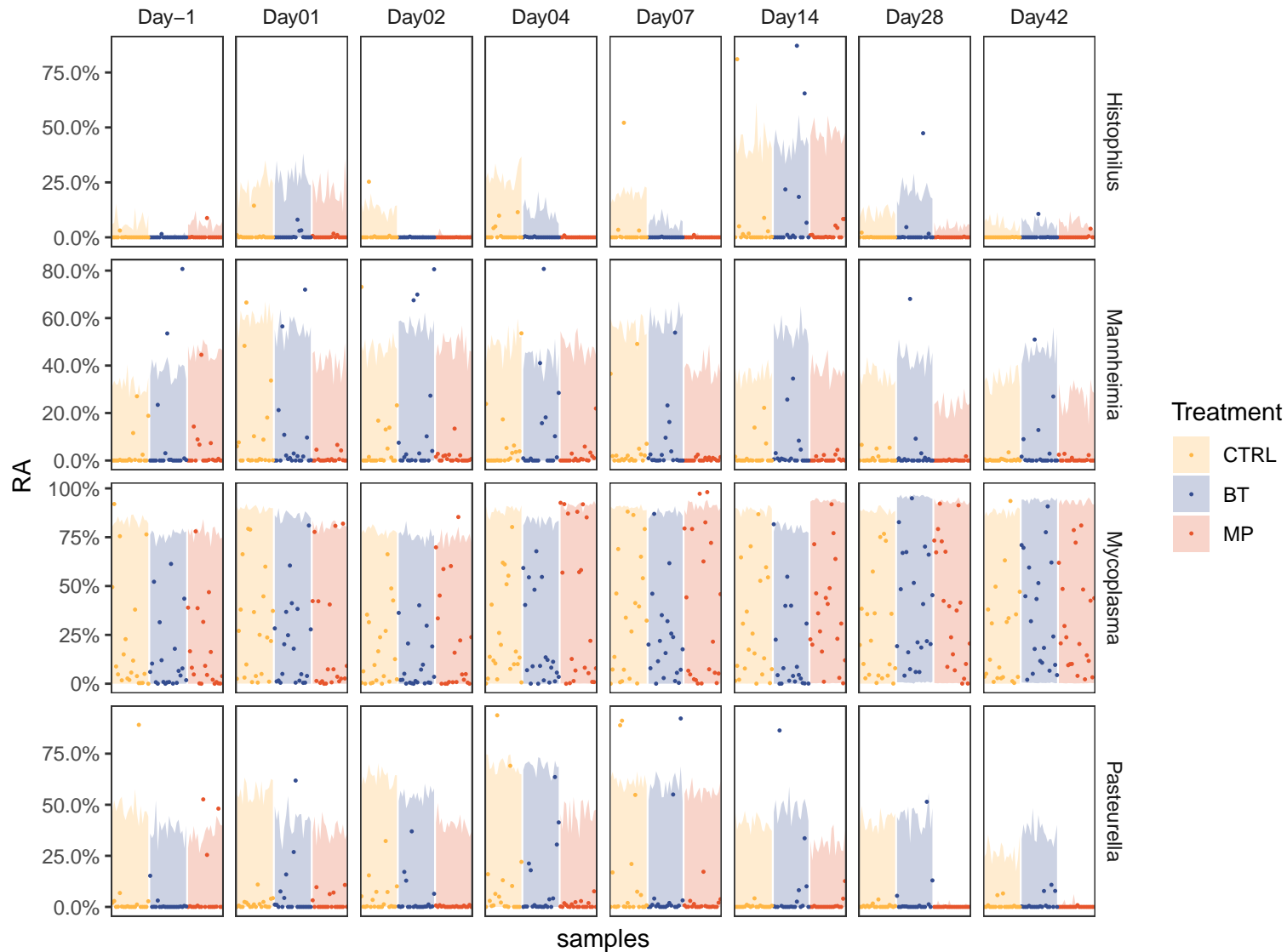

Supplement: FIG S4 [file msystems.01016-22-s0007.pdf]

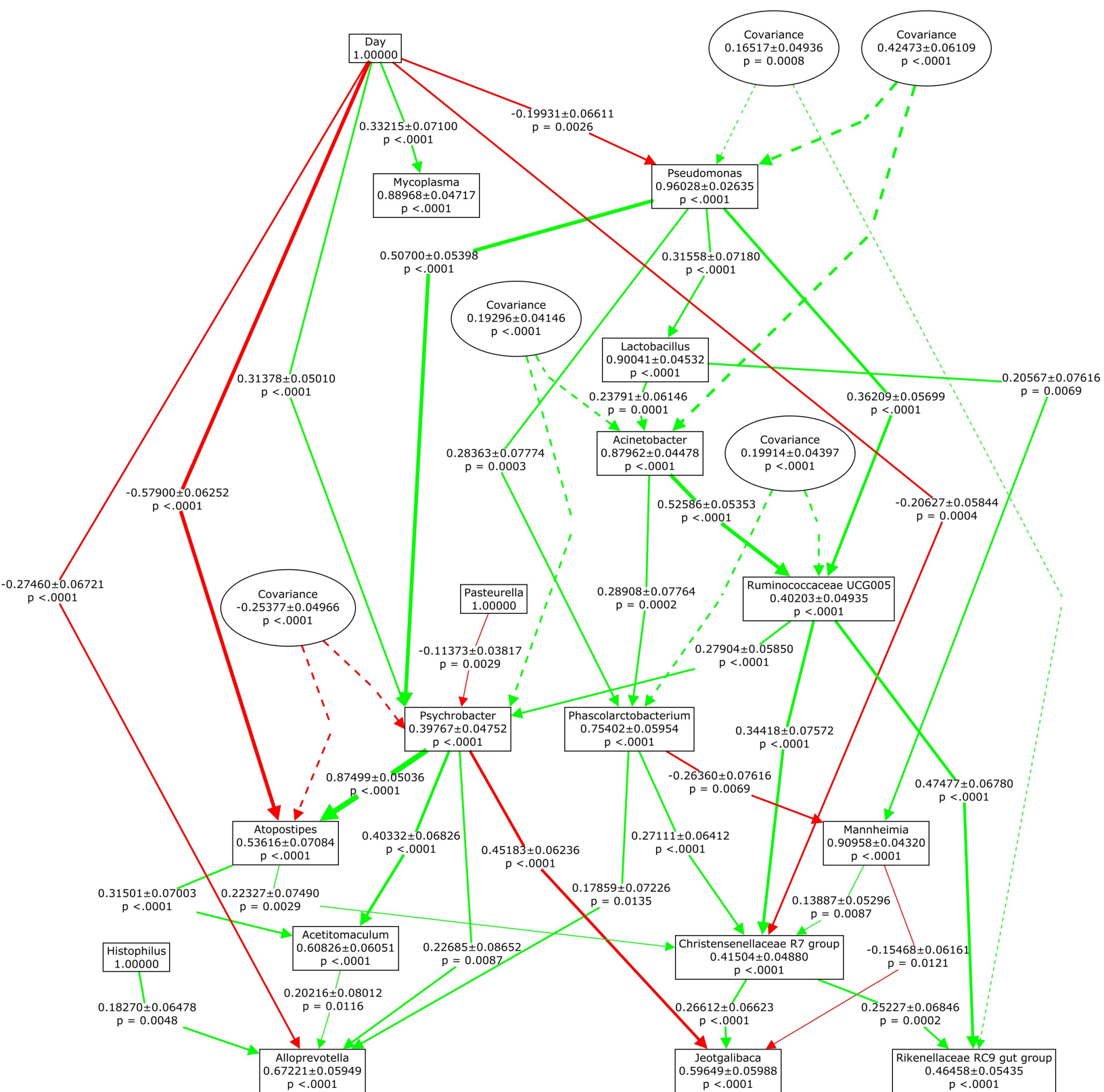

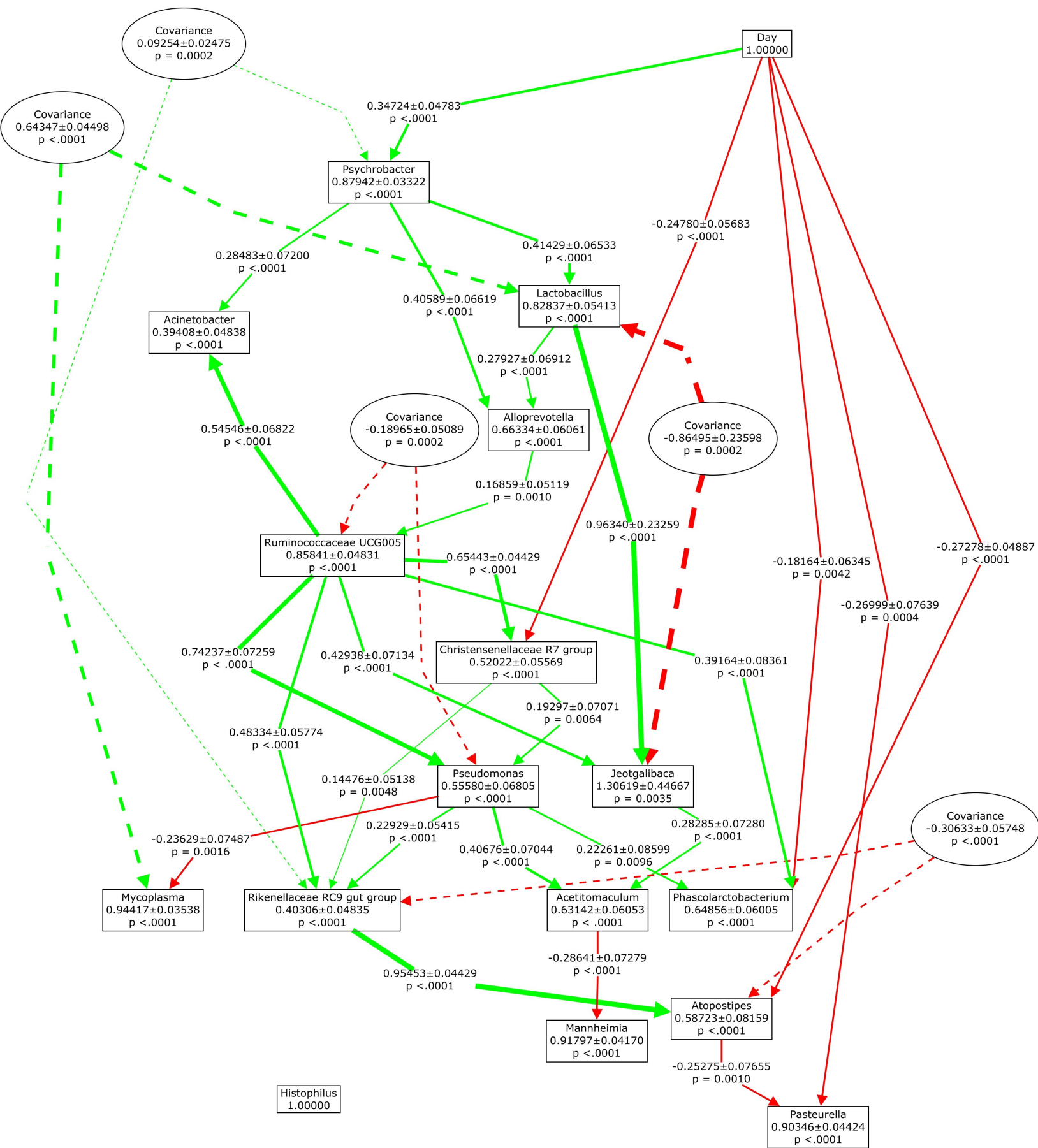

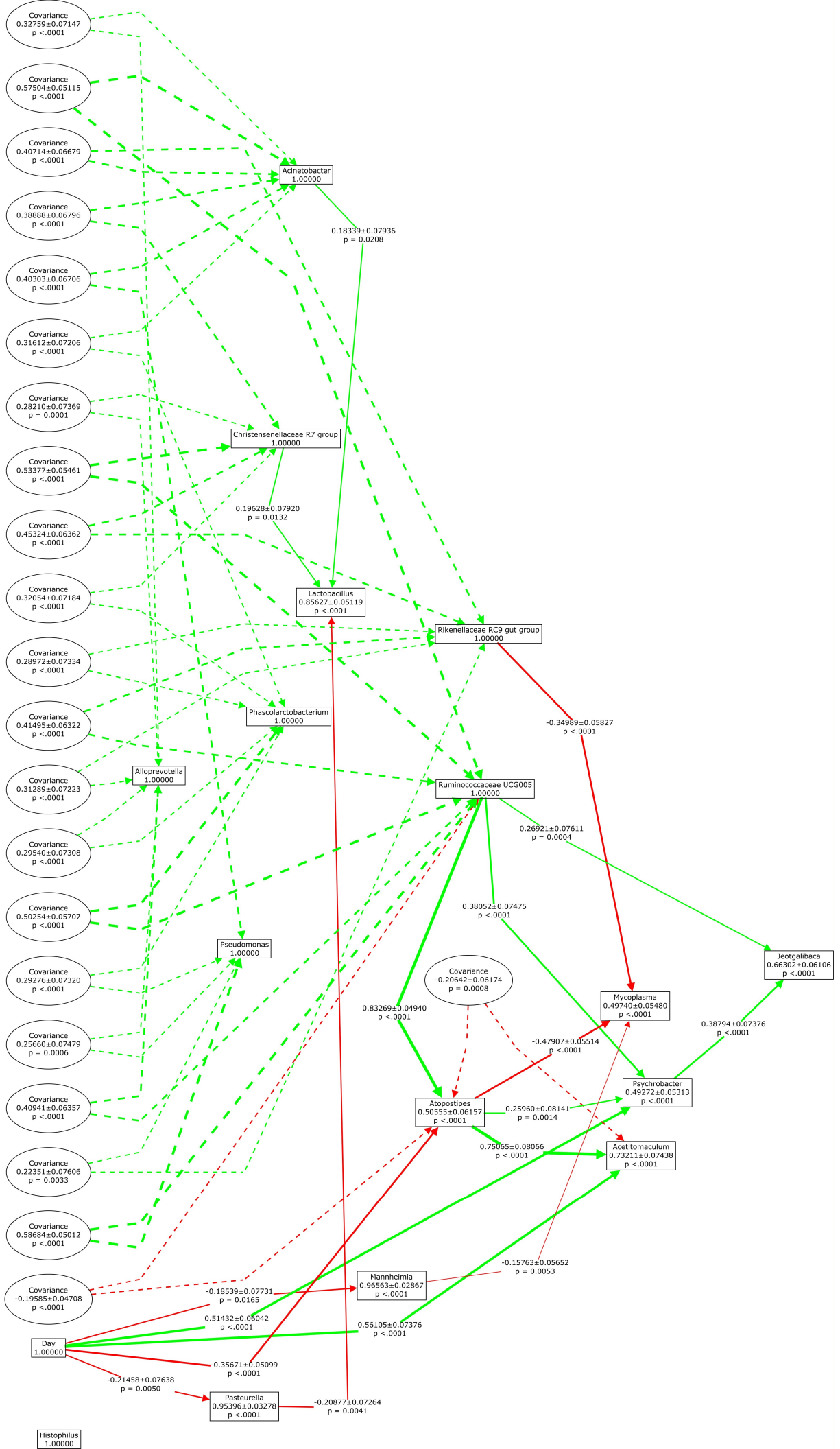

Supplement: FIG S5 [file msystems.01016-22-s0008.pdf]
