# Supplementary material for: A Single Intranasal Dose of Bacterial Therapeutics to Calves Confers Longitudinal Modulation of the Nasopharyngeal Microbiota: a Pilot Study
Source: mSystems. 2023 Mar 27;8(2):e01016-22. doi: 10.1128/msystems.01016-22 (PMC10134831; doi:10.1128/msystems.01016-22)
Supplement: TABLE S2 [file msystems.01016-22-s0002.docx]

**Supplementary table S2.** (Model 1, BT group).

|  | **Ace** | **Aci** | **Ato** | **Chr** | **Lac** | **Man** | **Pha** | **Pse** | **Psy** | **Rum** | **Day** | **His** | **Pas** |
| --- | --- | --- | --- | --- | --- | --- | --- | --- | --- | --- | --- | --- | --- |
| **Ace** | 0 | 0.10 ± 0.025 | 0.32 ± 0.07 | 0 | 0.24 ± 0.009 | 0 | 0 | 0.42 ± 0.044 | 0.68 ± 0.053 | 0.19 ± 0.042 | -0.05 ± 0.051 | 0 | -0.08 ± 0.026 |
|  |  | *p* < .0001 | *p* < .0001 |  | *p* = 0.0073 |  |  | *p* < .0001 | *p* < .0001 | *p* < .0001 | *p* = 0.3016 |  | *p* = 0.0034 |
| **Aci** | 0 | 0 | 0 | 0 | 0.24 ± 0.062 | 0 | 0 | 0.08 ± 0.026 | 0 | 0 | -0.02 ± 0.007 | 0 | 0 |
|  |  |  |  |  | *p* = 0.0001 |  |  | *p* = 0.0038 | |  | *p* = 0.0405 |  |  |
| **All** | 0.20 ± 0.081 | 0.11 ± 0.027 | 0.07 ± 0.029 | 0 | 0.03 ± 0.009 | 0 | 0.18 ± 0.072 | 0.28 ± 0.045 | 0.36 ± 0.072 | 0.10 ± 0.029 | -0.25 ± 0.068 | 0.18 ± 0.065 | -0.04 ± 0.016 |
|  | *p* = 0.0116 | *p* = 0.0001 | *p* = 0.0284 |  | *p* = 0.0076 |  | *p* = 0.0135 | *p* < .0001 | *p* < .0001 | *p* = 0.0005 | *p* = 0.0001 | *p* = 0.0048 | *p* = 0.0099 |
| **Ato** | 0 | 0.13 ± 0.031 | 0 | 0 | 0.03 ± 0.011 | 0 | 0 | 0.54 ± 0.042 | 0.88 ± 0.05 | 0.24 ± 0.052 | -0.41 ± 0.053 | 0 | -0.10± 0.034 |
|  |  | *p* < .0001 |  |  | *p* = 0.0064 |  |  | *p* < .0001 | *p* < .0001 | *p* < .0001 | *p* < .0001 |  | *p* = 0.0034 |
| **Chr** | 0 | 0.28 ± 0.044 | 0.22 ± 0.075 | 0 | 0.10 ± 0.025 | 0.14 ± 0.053 | 0.24 ± 0.064 | 0.34 ± 0.043 | 0.20 ± 0.067 | 0.40 ± 0.067 | -0.34 ± 0.055 | 0 | -0.02 ± 0.011 |
|  |  | *p* < .0001 | *p* = 0.0029 |  | *p* = 0.0002 | *p* = 0.0087 | *p* = 0.0002 | *p* < .0001 | *p* = 0.0033 | *p* < .0001 | *p* < .0001 |  | *p* = 0.0034 |
| **Jeo** | 0 | 0.15 ± 0.029 | 0.06 ± 0.025 | 0.27 ± 0.066 | 0.01 ± 0.02 | -0.12 ± 0.063 | 0.10 ± 0.03 | 0.37 ± 0.043 | 0.50 ± 0.058 | 0.23 ± 0.041 | -0.01 ± 0.045 | 0 | -0.06 ± 0.020 |
|  |  | *p* < .0001 | *p* = 0.0168 | *p* < .0001 | *p* = 0.5419 | *p* = 0.0632 | *p* = 0.0005 | *p* < .0001 | *p* < .0001 | *p* < .0001 | *p* = 0.904 |  | *p* = 0.0047 |
| **Lac** | 0 | 0 | 0 | 0 | 0 | 0 | 0 | 0.32 ± 0.072 | 0 | 0 | -0.06 ± 0.026 | 0 | 0 |
|  |  |  |  |  |  |  |  | *p* < .0001 |  |  | *p* = 0.0137 |  |  |
| **Man** | 0 | -0.08 ± 0.03 | 0 | 0 | 0.19 ± 0.076 | 0 | -0.26 ± 0.075 | -0.02 ± 0.039 | 0 | 0 | 0.003 ± 0.008 | 0 | 0 |
|  |  | *p* = 0.0118 |  |  | *p* = 0.0134 |  | *p* = 0.0004 | *p* = 0.6922 | |  | *p* = 0.6948 |  |  |
| **Myc** | 0 | 0 | 0 | 0 | 0 | 0 | 0 | 0 | 0 | 0 | 0.33 ± 0.071 | 0 | 0 |
|  |  |  |  |  |  |  |  |  |  |  | *p* < .0001 |  |  |
| **Pha** | 0 | 0.29 ± 0.078 | 0 | 0 | 0.07 ± 0.026 | 0 | 0 | 0.31 ± 0.075 | 0 | 0 | -0.06 ± 0.025 | 0 | 0 |
|  |  | *p* = 0.0002 |  |  | *p* = 0.0077 |  |  | *p* < .0001 |  |  | *p* = 0.0164 |  |  |
| **Pse** | 0 | 0 | 0 | 0 | 0 | 0 | 0 | 0 | 0 | 0 | -0.20 ± 0.066 | 0 | 0 |
|  |  |  |  |  |  |  |  |  |  |  | *p* = 0.0026 |  |  |
| **Psy** | 0 | 0.15 ± 0.035 | 0 | 0 | 0.04 ± 0.013 | 0 | 0 | 0.62 ± 0.043 | 0 | 0.28 ± 0.056 | 0.19 ± 0.057 | 0 | -0.11± 0.038 |
|  |  | *p* < .0001 |  |  | *p* = 0.0063 |  |  | *p* < .0001 |  | *p* < .0001 | *p* = 0.0008 |  | *p* = 0.0029 |
| **Rik** | 0 | 0.32 ± 0.044 | 0.06 ± 0.024 | 0.25 ± 0.069 | 0.08 ± 0.023 | 0.04 ± 0.016 | 0.06 ± 0.023 | 0.28 ± 0.039 | 0.05 ± 0.022 | 0.58 ± 0.054 | -0.12 ± 0.032 | 0 | -0.01 ± 0.003 |
|  |  | *p* < .0001 | *p* = 0.021 | *p* = 0.0002 | *p* = 0.0002 | *p* = 0.033 | *p* = 0.0095 | *p* < .0001 | *p* = 0.0216 | *p* < .0001 | *p* = 0.0001 |  | *p* = 0.0684 |
| **Rum** | 0 | 0.53 ± 0.054 | 0 | 0 | 0.13 ± 0.035 | 0 | 0 | 0.40 ± 0.056 | 0 | 0 | -0.08 ± 0.029 | 0 | 0 |
|  |  | *p* < .0001 |  |  | *p* = 0.0004 |  |  | *p* < .0001 |  |  | *p* = 0.0062 |  |  |

Genera**: Ace**, *Acetitomaculum*; **Aci**, *Acinetobacter;* **All***, Alloprevotella*; **Ato**, *Atopostipes*; **Chr**, *Christensenellaceae_R7_group*;**His**, *Histophilus*; **Jeo,** *Jeotgalibaca*; **Lac**, *Lactobacillus*; **Man**, *Mannheimia*; **Myc**, *Mycoplasma*; **Pas**, *Pasteurella*; **Pha**, *Phascolarctobacterium*; **Pse**, *Pseudomonas*; **Psy**, *Psychrobacter*; **Rik**, *Rikenellaceae_RC9_gut_group*; **Rum**, *Ruminococcaceae_UCG005*

**Supplementary table S2.** Continue (Model 2, CTRL group).

|  | **Ace** | **All** | **Ato** | **Chr** | **Jeo** | **Lac** | **Pse** | **Psy** | **Rik** | **Rum** | **Day** | **His** |
| --- | --- | --- | --- | --- | --- | --- | --- | --- | --- | --- | --- | --- |
| **Ace** | 0 | 0.08 ± 0.026 | 0 | 0.08 ± 0.032 | 0.28 ± 0.073 | 0.30 ± 0.100 | 0.41 ± 0.070 | 0.16 ± 0.037 | 0 | 0.48 ± 0.055 | 0.03 ± 0.019 | 0 |
|  |  | *p* = 0.0020 |  | *p* = 0.014 | *p* = 0.0001 | *p* = 0021 | *p* <.0001 | *p* <.0001 |  | *p* <.0001 | *p* = 0.0705 | |
| **Aci** | 0 | 0.09 ± 0.030 | 0 | 0 | 0 | 0.03 ± 0.011 | 0 | 0.33 ± 0.069 | 0 | 0.55 ± 0.068 | 0.12 ± 0.029 | 0 |
|  |  | *p* = 0.0024 |  |  |  | *p* = 0.0163 |  | p <.0001 |  | *p* <.0001 | *p* <.0001 |  |
| **All** | 0 | 0 | 0 | 0 | 0 | 0.28 ± 0.069 | 0 | 0.52 ± 0.057 | 0 | 0 | 0.18 ± 0.032 | 0 |
|  |  |  |  |  |  | *p* <.0001 |  | *p* <.0001 |  |  | *p* <.0001 |  |
| **Ato** | 0 | 0.13 ± 0.038 | 0 | 0.18 ± 0.49 | 0 | 0.04 ± 0.014 | 0.22 ± 0.052 | 0.07 ± 0.022 | 0.96 ± 0.044 | 0.74 ± 0.037 | -0.30 ± 0.050 | 0 |
|  |  | *p* = 0.0010 |  | *p* = 0.0002 |  | *p* = 0.0118 | *p* <.0001 | *p* = 0.0024 | *p* <.0001 | *p* <.0001 | *p* <.0001 |  |
| **Chr** | 0 | 0.11 ± 0.035 | 0 | 0.11 ± 0.0002 | 0 | 0.04 ± 0.012 | 0 | 0.06 ± 0.020 | 0 | 0.65 ± 0.044 | -0.23± 0.057 | 0 |
|  |  | *p* = 0.0014 |  |  |  | *p* = 0.0131 |  | *p* = 0.003 |  | *p* <.0001 | *p* <.0001 |  |
| **Jeo** | 0 | 0.07 ± 0.025 | 0 | 0 | 0 | 0.98 ± 0.230 | 0 | 0.44 ± 0.067 | 0 | 0.43 ± 0.071 | 0.15 ± 0.032 | 0 |
|  |  | *p* = 0.0040 |  |  |  | *p* <.0001 |  | *p* <.0001 |  | *p* <.0001 | *p* <.0001 |  |
| **Lac** | 0 | 0 | 0 | 0 | 0 | 0 | 0 | 0.41 ± 0.065 | 0 | 0 | 0.14 ± 0.03 | |
|  |  |  |  |  |  |  |  | *p* <.0001 |  |  | *p* <.0001 |  |
| **Man** | -0.29 ± 0.072 | -0.02 ± 0.010 | 0 | -0.02 ± 0.011 | -0.08 ± 0.030 | -0.084 ± 0.035 | -0.12 ± 0.037 | -0.04 ± 0.016 | 0 | -0.13 ± 0.039 | (-0.01 ± 0.006) | 0 |
|  | *p* < .0001 | *p* = 0.016 |  | *p* = 0.0386 | *p* = 0.0063 | *p* = 0.0162 | *p* = 0.0014 | *p* = 0.0048 |  | *p* = 0.0005 | *p* = 1017 |  |
| **Myc** | 0 | -0.03 ± 0.015 | 0 | -0.45 ± 0.022 | 0 | -0.01 ± 0.005 | -0.24 ± 0.075 | -0.02± 0.016 | 0 | -0.21± 0.067 | 0.01 ± 0.006 | 0 |
|  |  | *p* = 0.0238 |  | *p* = 0.0395 |  | *p* = 0.05 | *p* = 0.0016 | *p* = 0.0303 |  | *p* = 0.002 | *p* =0.379 |  |
| **Pas** | 0 | -0.03 ± 0.014 | -0.25 ± 0.076 | -0.45 ± 0.019 | 0 | -0.01 ± 0.004 | -0.06 ± 0.021 | -0.02 ± 0.007 | -0.24 ± 0.074 | -0.19 ± 0.058 | -0.20 ± 0.075 | 0 |
|  |  | *p* = 0.02 | *p* = 0.001 | *p* = 0.0144 |  | *p* = 0.0451 | *p* = 0.001 | *p* = 0.0263 | *p* = 0.0011 | *p* = 0.0012 | *p* = 0.0092 | |
| **Pha** | 0 | 0.10 ± 0.031 | 0 | 0.04 ± 0.023 | 0 | 0.03 ± 0.011 | 0.22 ± 0.086 | 0.05 ± 0.018 | 0 | 0.59 ± 0.055 | -0.17 ± 0.064 | 0 |
|  |  | *p* = 0.0017 |  | *p* = 0.0609 |  | *p* = 0.0141 | *p* = 0.001 | *p* = 0.0035 | 1 | *p* <.0001 | *p* = 0.0063 | |
| **Pse** | 0 | 0.15 ± 0.045 | 0 | 0.19 ± 0.071 | 0 | 0.04 ± 0.016 | 0 | 0.08 ± 0.025 | 0 | 0.87 ± 0.05 | -0.02 ± 0.023 | 0 |
|  |  | *p* = 0.0011 |  | *p* = 0.0064 |  | *p* = 0.0141 |  | *p* = 0.0024 |  | *p* <.0001 | *p* = 0.3606 | |
| **Psy** | 0 | 0 | 0 | 0 | 0 | 0 | 0 | 0 | 0 | 0 | 0.35 ± 0.048 | 0 |
|  |  |  |  |  |  |  |  |  |  |  | *p* <.0001 |  |
| **Rik** | 0 | 0.13 ± 0.040 | 0 | 0.19 ± 0.051 | 0 | 0.04 ±0.015 | 0.23 ± 0.054 | 0.07 ± 0.022 | 0 | 0.78 ± 0.034 | -0.02 ± 0.019 | 0 |
|  |  | *p* = 0.0009 |  | *p* = 0.0002 |  | *p* = 0.0114 | *p* <.0001 | *p* = 0.0021 |  | *p* <.0001 | *p* = 0.2266 | |
| **Rum** | 0 | 0.17 ± 0.051 | 0 | 0 | 0 | 0.05 ± 0.019 | 0 | 0.09 ± 0.029 | 0 | 0 | 0.03 ± 0.011 | 0 |
|  |  | *p* = 0.0001 |  |  |  | *p* = 0.0117 |  | *p* = 0.0021 |  |  | *p* = 0.0073 | |

**Supplementary table S2.** Continue (Model 3, MP group).

|  | **At** | **Ma** | **Pa** | **Ps** | **Ac** | **Al** | | **Ch** | **Day** | **Hi** | **Ph** | **Ps** | **Ri** | **Ru** |
| --- | --- | --- | --- | --- | --- | --- | --- | --- | --- | --- | --- | --- | --- | --- |
| **Ac** | 0.75 ± 0.080 | 0 | 0 | 0 | 0 | 0 | 0 | | 0.29 ± 0.063 | 0 | 0 | 0 | 0 | 0.6251 ± 0.068 |
|  | *p* <.0001 |  |  |  |  |  |  | | *p* < .0001 |  |  |  |  | *p* < .0001 |
| **At** | 0 | 0 | 0 | 0 | 0 | 0 | 0 | | -0.36 ± 0.051 | 0 | 0 | 0 | 0 | 0.83 ± 0.049 |
|  |  |  |  |  |  |  |  | | *p* < .0001 |  |  |  |  | *p* < .0001 |
| **Je** | 0.10 ± 0.037 | 0 | 0 | 0.39 ± 0.074 | 0 | 0 | 0 | | 0.16 ± 0.039 | 0 | 0 | 0 | 0 | 0.50 ± 0.060 |
|  | *p*= 0.0068 |  |  | *p* <.0001 |  |  |  | | *p* < .0001 |  |  |  |  | *p* < .0001 |
| **La** | 0 |  | -0.21 ± 0.073 | 0 | 0.18 ± 0.079 | 0 | 0.20 ± 0.079 | | 0.045 ± 0.023 | 0 | 0 | 0 | 0 | 0 |
|  |  |  | *p* = 0.0041 |  | *p* = 0.0208 |  | *p* = 0.0132 | | *p* = 0.0471 |  |  |  |  |  |
| **Ma** | 0 | 0 | 0 | 0 | 0 | 0 | 0 | | -0.19 ± 0.077 | 0 | 0 | 0 | 0 | 0 |
|  |  |  |  |  |  |  |  | | *p* = 0.0165 |  |  |  |  |  |
| **My** | -0.48 ± 0.055 | -0.16 ± 0.057 | 0 | 0 | 0 | 0 | 0 | | 0.02 ± 0.035 | 0 | 0 | 0 | -0.35 ± 0.058 | -0.40 ± 0.053 |
|  | *p* <.0001 | *p* = 0.0053 |  |  |  |  |  | | *p* < .0001 |  |  |  | *p* < .0001 | *p* < .0001 |
| **Pa** | 0 | 0 | 0 | 0 | 0 | 0 | 0 | | -0.22 ± 0.076 | 0 | 0 | 0 | 0 | 0 |
|  |  |  |  |  |  |  |  | | *p* = 0.005 |  |  |  |  |  |
| **Ps** | 0.26 ± 0.081 | 0 | 0 | 0 | 0 | 0 | 0 | | 0.42 ± 0.055 | 0 | 0 | 0 | 0 | 0.60 ± 0.051 |
|  | *p* = 0.0014 |  |  |  |  |  |  | | *p* < .0001 |  |  |  |  | *p* < .0001 |
