# Supplementary material for: A Single Intranasal Dose of Bacterial Therapeutics to Calves Confers Longitudinal Modulation of the Nasopharyngeal Microbiota: a Pilot Study
Source: mSystems. 2023 Mar 27;8(2):e01016-22. doi: 10.1128/msystems.01016-22 (PMC10134831; doi:10.1128/msystems.01016-22)
Supplement: TABLE S3 [file msystems.01016-22-s0003.docx]

**Supplementary table S3**

|  | **Model 1 (BT)** | **Model 2 (CTRL)** |
| --- | --- | --- |
| *Acetitomaculum* | 0.0237 ± 0.008837  *p* = 0.007316 | 0.2949 ± 0.1018  *p* = 0.003762 |
| *Acinetobacter* | 0.2379 ± 0.0615  *p* = 0.000108 | 0.0257 ± 0.0107  *p* = 0.0166 |
| *Alloprevotella* | 0.025 ± 0.009363  *p* = 0.007598 | 0.2794 ± 0.071  *p* <.0001 |
| *Atopostipes* | 0.0305 ± 0.0112,  *p* = 0.006419 | 0.0348 ± 0.0138  *p* = 0.0116 |
| *Christensenellaceae_R7_group* | 0.0946 ± 0.0251  *p* 0.00016 | 0.0307 ± 0.0124  *p* = 0.0136 |
| *Jeotgalibaca* | 0.0119 ± 0.0196  *p* = 0.5419 | 0.9835 ± 0.2532  *p* = 0.000102 |
| *Mannheimia* | 0.1875 ± 0.0758  *p* = 0.0134 | -0.0844 ± 0.0367  *p* = 0.0216 |
| *Mycoplasma* | 0 | -0.00966 ± 0.004982  *p* = 0.0525 |
| *Pasteurella* | 0 | -0.0883 ± 0.004454  *p* = 0.0474 |
| *Phascolarctobacterium* | 0.0688 ± 0.0258  *p* = 0.007703 | 0.0275 ± 0.0113  *p* = 0.0149 |
| *Pseudomonas* | 0 | 0.0409 ± 0.0163  *p* = 0.0123 |
| *Psychrobacter* | 0.0349 ± 0.0128  *p* = 0.006275 | 0 |
| *Rikenellaceae_RC9_gut_group* | 0.0833 ± 0.0225  *p* = 0.00021 | 0.0366 ± 0.0145  *p* = 0.0116 |
| *Ruminococcaceae_UCG005* | 0.1251 ± 0.0352  *p* = 0.00038 | 0.047 ± 0.0186  *p* = 0.0115 |
